# Screening of Chemical Libraries for New Antifungal Drugs against Aspergillus fumigatus Reveals Sphingolipids Are Involved in the Mechanism of Action of Miltefosine

**DOI:** 10.1128/mBio.01458-21

**Published:** 2021-08-10

**Authors:** Thaila Fernanda dos Reis, Maria Augusta Crivelente Horta, Ana Cristina Colabardini, Caroline Mota Fernandes, Lilian Pereira Silva, Rafael Wesley Bastos, Maria Vitória de Lazari Fonseca, Fang Wang, Celso Martins, Márcio L. Rodrigues, Cristina Silva Pereira, Maurizio Del Poeta, Koon Ho Wong, Gustavo H. Goldman

**Affiliations:** a Faculdade de Ciências Farmacêuticas de Ribeirão Preto, Universidade de São Paulo, Ribeirão Preto, Brazil; b MicroControl Innovation Ltd., Ribeirão Preto, São Paulo, Brazil; c Department of Microbiology and Immunology, Stony Brook University, Stony Brook, New York, USA; d Faculty of Health Sciences, University of Macau, Taipa, Macau, SAR, China; e Instituto de Tecnologia Química e Biológica António Xavier, Universidade Nova de Lisboa (ITQB NOVA), Oeiras, Portugal; f Instituto Carlos Chagas (ICC), Fundação Oswaldo Cruz–Fiocruz, Curitiba, Brazil; g Instituto de Microbiologia Paulo de Góes, Universidade Federal do Rio de Janeiro (UFRJ), Rio de Janeiro, Brazil; h Veteran Administration Medical Center, Northport, New York, USA; i MicroRid Technologies Inc., Dix Hills, New York, USA; j Division of Infectious Diseases, School of Medicine, Stony Brook University, New York, USA; k Institute of Translational Medicine, Faculty of Health Sciences, University of Macau, Avenida da Universidade, Taipa, Macau, SAR, China; l MoE Frontiers Science Center for Precision Oncology, University of Macau, Taipa, Macau, SAR, China; Yonsei University

**Keywords:** *Aspergillus fumigatus*, drug repurposing, miltefosine, sphingolipids, transcription factor

## Abstract

Aspergillus fumigatus is an important fungal pathogen and the main etiological agent of aspergillosis, a disease characterized by a noninvasive process that can evolve to a more severe clinical manifestation, called invasive pulmonary aspergillosis (IPA), in immunocompromised patients. The antifungal arsenal to threat aspergillosis is very restricted. Azoles are the main therapeutic approach to control IPA, but the emergence of azole-resistant A. fumigatus isolates has significantly increased over recent decades. Therefore, new strategies are necessary to combat aspergillosis, and drug repurposing has emerged as an efficient and alternative approach for identifying new antifungal drugs. Here, we used a screening approach to analyze A. fumigatus *in vitro* susceptibility to 1,127 compounds. A. fumigatus was susceptible to 10 compounds, including miltefosine, a drug that displayed fungicidal activity against A. fumigatus. By screening an A. fumigatus transcription factor null library, we identified a single mutant, which has the *smiA* (sensitive to miltefosine) gene deleted, conferring a phenotype of susceptibility to miltefosine. The transcriptional profiling (RNA-seq) of the wild-type and Δ*smiA* strains and chromatin immunoprecipitation coupled to next-generation sequencing (ChIP-Seq) of an SmiA-tagged strain exposed to miltefosine revealed genes of the sphingolipid pathway that are directly or indirectly regulated by SmiA. Sphingolipid analysis demonstrated that the mutant has overall decreased levels of sphingolipids when growing in the presence of miltefosine. The identification of SmiA represents the first genetic element described and characterized that plays a direct role in miltefosine response in fungi.

## INTRODUCTION

Fungi are widespread in nature, surviving as saprophytic organisms or associated with animals and plants, where they can behave as commensal or opportunistic organisms. In humans, pathogenic fungi can cause both superficial and invasive infections, giving rise to the death of millions of people annually (1–3). Cryptococcus, *Candida*, Aspergillus, and Pneumocystis species are responsible for the most representative invasive fungal infections (1), showing death rates as high as those of tuberculosis and malaria (2, 4, 5). The levels of mortality are dependent on host immune system integrity, being particularly important for immunocompromised patients (6–8). These individuals comprise a risk group that is expanding quickly due to the increasing number of immune-deficient patients who underwent transplant or chemotherapy and patients under therapy with high dosage of corticosteroids (9–11).

Aspergillus spp. cause a group of diseases collectively named aspergillosis, and their development occurs after the inhalation of conidia dispersed in the environment (12). In immunocompetent patients, the development of aspergillosis is mainly characterized by noninvasive diseases, including aspergilloma, chronic necrotizing pulmonary aspergillosis, chronic cavitary pulmonary aspergillosis, and chronic fibrotic pulmonary aspergillosis, which together are defined as chronic pulmonary aspergillosis (12–16). Invasive pulmonary aspergillosis (IPA) is an important clinical manifestation caused by Aspergillus spp., presenting high levels of mortality in immunocompromised patients (1, 17). IPA is the most common invasive fungal infection in recipients of both hematopoietic stem cells and solid-organ transplants (1, 17). In this group of high-risk patients for IPA, A. fumigatus represents the major cause of the disease, reaching up to 90% of mortality (9–12, 18).

Very few classes of antifungal drugs are available for IPA treatment, such as polyenes (amphotericin B), azoles (itraconazole, posaconazole, voriconazole, and isavuconazole), and echinocandins (caspofungin) (19–22). Although both amphotericin B and echinocandins can be used to treat IPA, these drugs have clinical limitations. Amphotericin B shows high levels of nephrotoxicity and side effects, while echinocandins are not fully recommended as monotherapy for IPA (9, 13, 23–25). So far, the administration of triazoles is the first therapeutic approach applied to control A. fumigatus infections showing the most prominent usage in the medical field (13, 26). Among them, itraconazole (introduced in 1990s), voriconazole (introduced in 2002), and posaconazole (introduced in 2006) are the most common drugs utilized for the treatment of aspergillosis (27). Voriconazole is the primary treatment against IPA, followed by liposomal amphotericin B (L-AMB) and echinocandins, which are recommended as a second-line therapy (13, 26, 28). Moreover, the activity of isavuconazole, a new extended-spectrum triazole drug, has been recently tested against Aspergillus (29–32).

The number of azole-resistant A. fumigatus clinical isolates has dramatically increased over recent decades and has become a major concern (28, 33–38). Additionally, azoles are also used in agriculture to combat plant-pathogenic fungi, and, recently, its usage for agricultural purposes has been linked to the emergence of azole-resistant isolates among human fungal pathogens (33, 39–42). Therefore, the emergence of global resistance to currently available antifungals agents represents a significant threat to immunosuppressed patients, as the current arsenal of antifungal drugs is very limited.

This situation highlights the need to understand the mechanisms of drug resistance and tolerance and the search for novel antifungal agents (43, 44). As few antifungal compounds are coming to market because their development is time-consuming and expensive, repositioning or repurposing drugs that are already licensed is an interesting and faster opportunity for the identification of novel antifungal agents (45–47). By using the repurposing strategy, many compounds have already been identified as new potential drugs against several diseases, including parasitosis, protozooses, and mycoses (45, 47–52). Here, we screened two chemical collections to analyze A. fumigatus *in vitro* susceptibility to compounds present in two compound libraries. The first library has active compounds against neglected diseases (The Pathogen Box), while the second one includes drugs previously approved for use against human diseases (National Institutes of Health [NIH] clinical collection [NCC]). We showed here that A. fumigatus was susceptible to at least 10 different compounds from the two libraries. One of these compounds, miltefosine, a drug mainly used in the treatment of visceral and cutaneous leishmaniasis (53, 54), demonstrated fungicidal activity against A. fumigatus. Aiming to get more insights about the mechanism of action of miltefosine, we screened an A. fumigatus transcription factor null mutant library (484 null mutants) and identified a single mutant highly sensitive to miltefosine. The gene deleted in this mutant was named *smiA* (sensitive to miltefosine). A combination of transcriptome sequencing (RNA-seq) and chromatin immunoprecipitation coupled to next-generation sequencing (ChIP-seq) studies revealed differentially expressed genes directly or indirectly regulated by SmiA. The sphingolipid (SL) profiling of the wild-type and the Δ*smiA* strains exposed to miltefosine revealed that the mutant has overall lower levels of sphingolipids than the wild type. Our results suggest that miltefosine displays antifungal activity against A. fumigatus by directly interfering in the sphingolipid biosynthetic pathway.

## RESULTS

### Screening of the Pathogen Box and NIH clinical library.

In order to find known compounds that are active against A. fumigatus, we tested its susceptibility to two chemical drug libraries, the Pathogen Box (containing 400 compounds; see https://www.mmv.org/mmv-org) and the National Institutes of Health (NIH) clinical collection (NCC) (containing 727 compounds; see https://pubchem.ncbi.nlm.nih.gov/source/NIH%20Clinical%20Collection) through MIC assays. In total, combining both libraries, 1,127 compounds were assessed by using MIC values up to 25 μM. A. fumigatus was susceptible to four known antifungal agents present in these collections (posaconazole, difenoconazole, bitertanol, and amphotericin B; MIC values of 5 μM, 5 μM, 5 μM and 10 μM, respectively). These results supported the reliability of the screening approach. A. fumigatus was also susceptible to other compounds, with MIC values ranging from 1.56 to 25 μM (Table 1). In Table 1, we describe the compound name, the MIC detected in our screening, the current usage purpose (description), and the mode of action (if known) for the 10 compounds. These compounds include (i) two azole salts, econazole and oxiconazole, expected to inhibit A. fumigatus growth to some extent; (ii) fluvastatin, a statin drug class used for hypercholesterolemia treatment; (iii) mesoridazine, a piperidine neuroleptic drug; (iv) cisapride, a parasympathomimetic drug acting as a serotonin 5-HT_4_ agonist; (v) indinavir sulfate, a protease inhibitor used in anti-HIV cocktails; (vi) enalaprilat, an angiotensin-converting enzyme inhibitor; (vii) vincristine sulfate, an inhibitor of microtubule formation in the mitotic spindle; (viii) iodoquinol, an anti-amoebiasis agent with an unknown mechanism of action; and (ix) miltefosine, an anti-*Leishmania* compound with an unknown mechanism of action (Table 1).

**TABLE 1:** MIC values for NIH clinical collection and Pathogen Box compounds against A. fumigatus

Compound	MIC (μM)	Description	Mode of action	Reference
Econazole nitrate	12.5	Broad-spectrum antimycotic agent	Inhibits ergosterol biosynthesis	120
Fluvastatin	25	Statin drug class used for hypercholesterolemia treatment; demonstrated antifungal activity against some fungal species	Blocks ergosterol biosynthesis by inhibition of farnesyl pyrophosphate production	121
Mesoridazine	3.12	Piperidine neuroleptic drug used for the treatment of schizophrenia, organic brain disorders, alcoholism, and psychoneuroses	Acts indirectly on reticular formation, whereby neuronal activity into reticular formation is reduced without affecting its intrinsic ability to activate the cerebral cortex	122
Cisapride	1.56	Gastroprokinetic agent, increases motility in the upper gastrointestinal tract	Parasympathomimetic acting as a serotonin 5-HT_4_ agonist	123
Oxiconazole nitrate	25	Salt form of oxiconazole with antifungal activity	Inhibits ergosterol biosynthesis	124
Indinavir sulphate	6.25	Antiretroviral protease inhibitor used in the therapy and prevention of HIV infection and AIDS	Protease inhibitor	125
Enalaprilat	25	Used in the treatment of hypertension	Angiotensin-converting enzyme inhibitor	126
Vincristine sulfate	25	Used in cancer chemotherapy	Inhibits microtubule formation in mitotic spindle salt of a natural alkaloid with antimitotic and antineoplastic activities	127
Iodoquinol	2	Antiprotozoal agent used as an amebicide drug	Unknown	128
Miltefosine	10	Antiprotozoal, bactericidal and antifungal agent	Unknown	129

**FIG 1:**
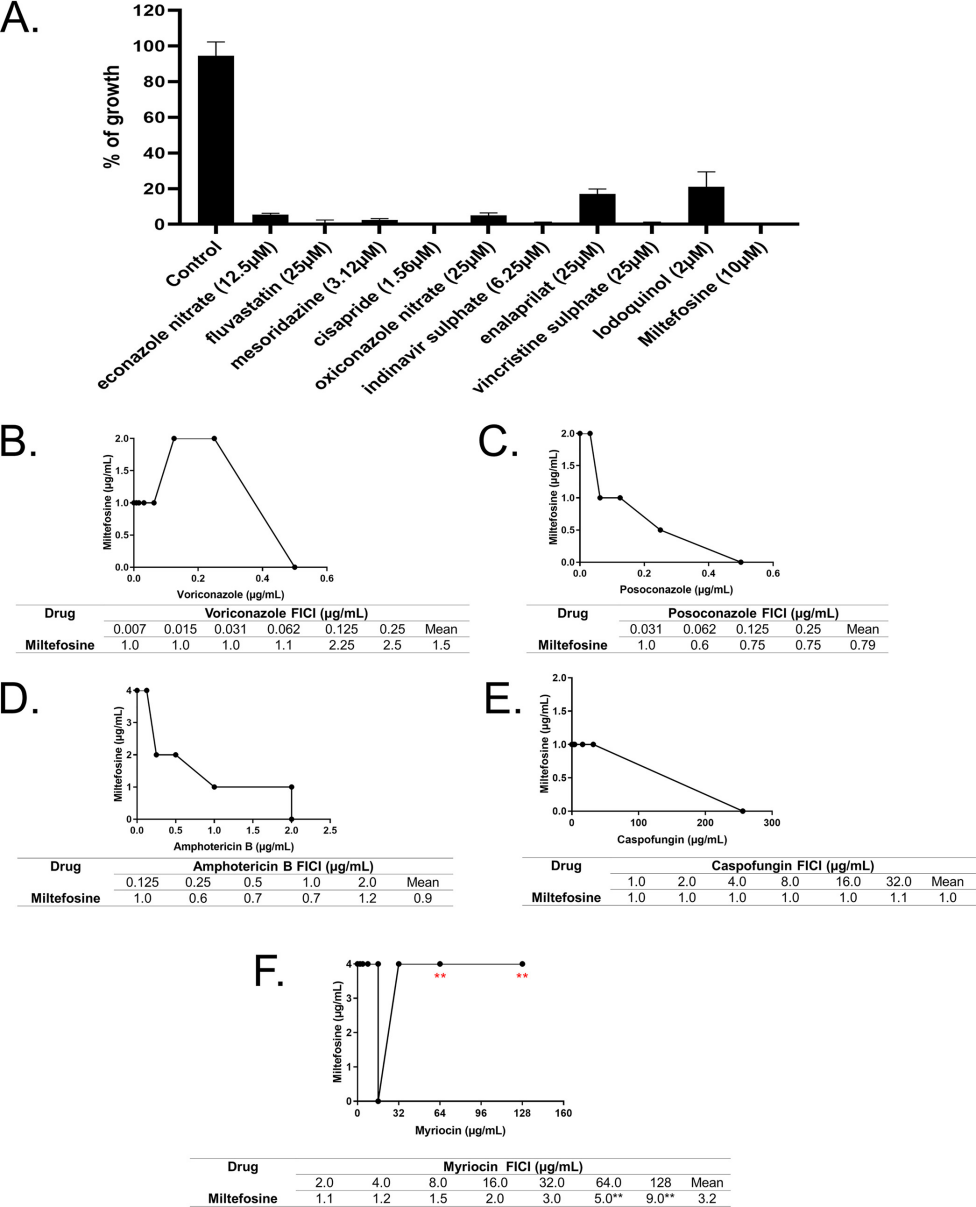
Miltefosine is a potential new anti-aspergillosis compound and shows its interaction with the sphingolipid inhibitor myriocin. (A) Screening of chemical libraries reveals potential new anti-aspergillosis compounds. (B) Interaction between miltefosine and posaconazole. (C) Interaction between miltefosine and voriconazole. (D) Interaction between miltefosine and amphotericin B. (E) Interaction between miltefosine and caspofungin. (F) Interaction between miltefosine and myriocin.

To determine if these compounds are fungicidal or fungistatic, A. fumigatus conidial viability was tested after 48 h of exposure to each compound at its corresponding MIC (Fig. 1A). Five compounds (fluvastatin, cisapride, indinavir sulfate, vincristine sulfate, and miltefosine) had a 100% fungicidal while six had a fungistatic mechanism of action, with 80% to 95% conidial killing at the MIC (Fig. 1A).

These results suggest that some of these compounds are fungicidal and can act directly in specific A. fumigatus cell targets, while others (like cisapride and enalaprilat) could be a lead compound in antifungal drug discovery.

### Miltefosine displays antagonistic interaction with myriocin, a sphingosine biosynthesis inhibitor.

We decided to investigate miltefosine in more detail because it is a fungicidal drug with an unknown mechanism of action. The combination between drugs is commonly used in clinical practice aiming to potentialize the antifungal effect of the drugs (55). Furthermore, the combination assay can help unravel the mechanism of action of the drugs and how this may vary according to the concentration (56). To check if miltefosine has any interaction with other antifungal drugs, we combined this compound (ranging from 0.001 to 8.0 μg/ml; MIC value of 10 μM corresponds to 4 μg/ml) with different antifungal drugs (Fig. 1B to 1E). Miltefosine was combined with posaconazole (0.03 to 2.0 μg/ml), voriconazole (0.0007 to 0.5 μg/ml), amphotericin B (0.06 to 4.0 μg/ml), and caspofungin (4.0 to 256.0 μg/ml) (Fig. 1B to 1E). Using the checkerboard microdilution method, the interaction between miltefosine and the other compounds was determined through the fractional inhibitory concentration index (FICI). The interaction between the drugs was classified as synergistic (FICI ≤ 0.5), indifferent (0.5 < FICI ≤ 4.0) or antagonistic (FICI > 4.0) (57). Under the assayed conditions, the FICI index varied from 1 to 2.5 in the combination of voriconazole and miltefosine, 1 to 0.6 between posaconazole and miltefosine, 0.6 to 1.2 between amphotericin B and miltefosine, and 1.0 to 1.1 between caspofungin and miltefosine. These data show that the addition of miltefosine did not affect the antifungal effects of the tested clinical antifungals against A. fumigatus, indicating that there is no interaction between them.

There is evidence in the literature showing that miltefosine can affect the sphingolipid metabolism in trypanosomatids (58, 59). To check if miltefosine could display any interaction with drugs that affect cellular lipid biosynthesis, we combined different concentrations of miltefosine (0.001 to 8.0 μg/ml) and myriocin (2.0 to 128 μg/ml) (Fig. 1F), an inhibitor of serine palmitoyltransferase, the first step in sphingosine biosynthesis (60). At low concentrations of the drugs, indifferent interaction was observed. Interestingly, at high concentrations, myriocin impaired the antifungal effects of miltefosine against A. fumigatus, demonstrating an antagonistic effect between these compounds (Fig. 1F). Considering that myriocin has only a single target identified, this result suggests the existence of a component of the sphingolipid pathway important to the antifungal effect of miltefosine.

### SmiA is the major transcription factor that mediates miltefosine response in A. fumigatus.

**FIG 2:**
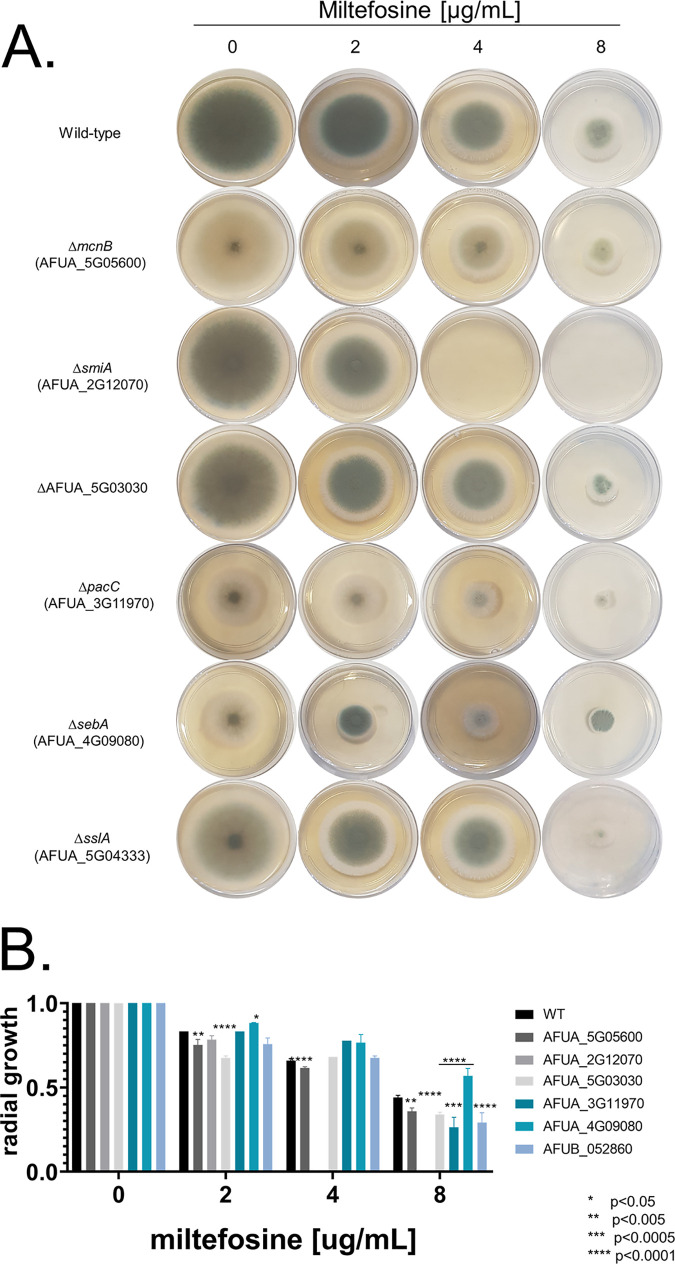
Radial growth of transcription factor (TF) null mutants in the presence of miltefosine. (A) A total of 1 × 10^5^ conidia of each species was inoculated on MM supplemented or not with increasing concentrations of miltefosine. Plates were incubated for 3 days at 37°C. (B) Quantification of the results obtained in panel A. For each strain, three independent experiments were realized, and the graphic shows the means ± standard deviations.

**FIG 3:**
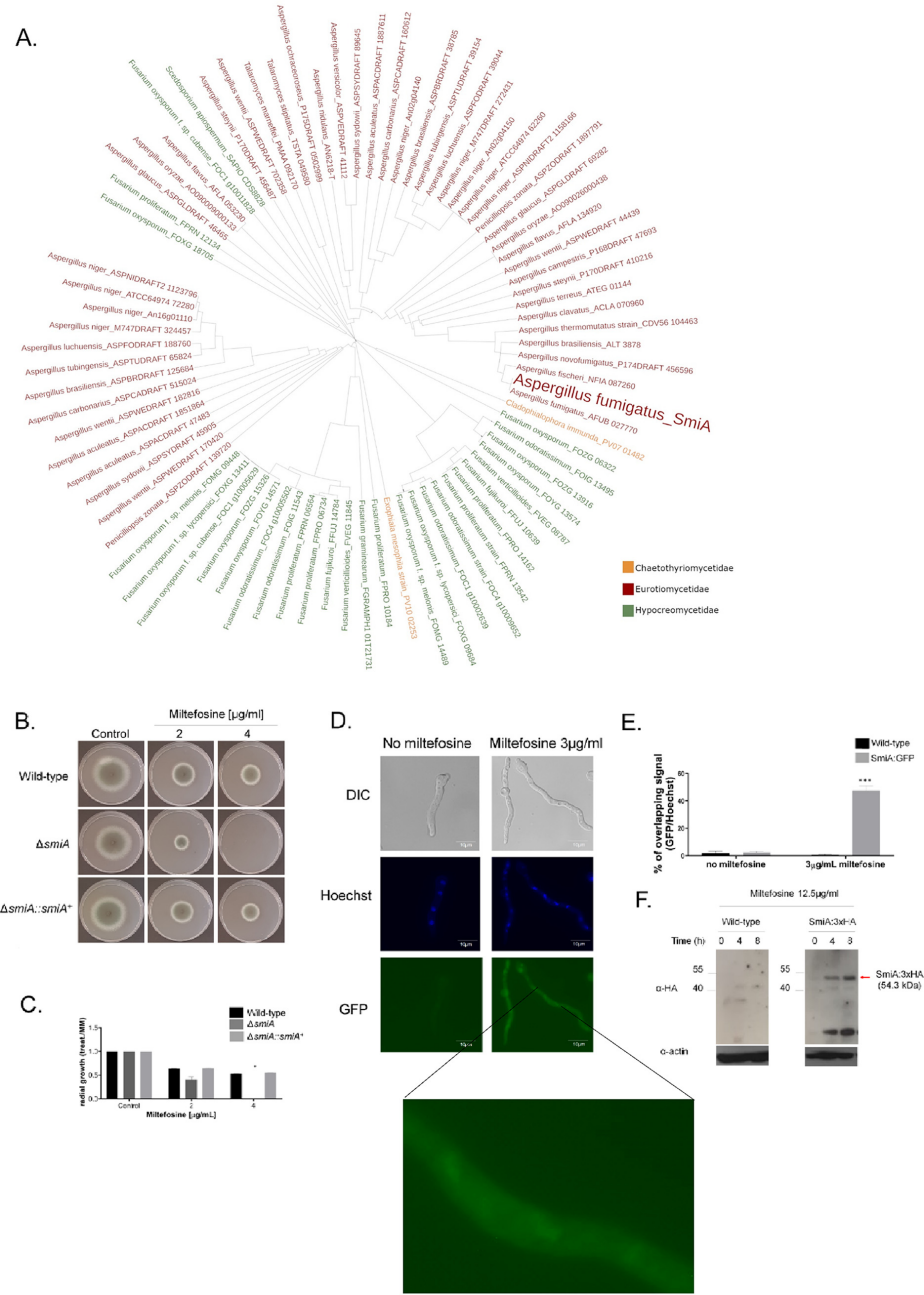
Molecular characterization of *smiA*. (A) The phylogenetic distribution of SmiA across fungal classes and genomes. Orthologs are determined using orthoMCL algorithm on FungiDB (www.fungidb.org). Sequences were aligned through pairwise Mercator (XX) analysis combined with Clustal Omega (YY). The phylogenetic tree was visualized with iTol v6 (ZZ). (B) Growth phenotypes of the wild-type, Δ*smiA*, and Δ*smiA*::*smiA*^+^ strains grown for 3 days on solid MM supplemented with increasing concentrations of miltefosine. (C) Graphical quantification of fungal growth presented in panel B. The results are averages ± standard deviations from three repetitions. (D) SmiA-GFP translocates to the nucleus under exposure to miltefosine. (E) Graphical quantification of SmiA-GFP location shown in panel D. The results are averages ± standard deviations from three repetitions of 30 germlings for each repetition. (F) Western blot showing the SmiA-HA expression after 0, 4, and 8 h of incubation with 12.5 μg/ml miltefosine. Anti-HA antibody was used to detect the recombinant protein. Anti-actin antibody was used as a loading control. Statistical analysis was performed using one-tailed, paired *t* tests for comparisons to the control condition (*, *P* < 0.05; ***, *P* < 0.001).

To assess if there are transcriptional programs modulating the tolerance response to miltefosine, a library of 484 A. fumigatus transcription factor (TF) null mutants (61) was screened for sensitivity to miltefosine (0.001 to 8.0 μg/ml). A primary screening using 96-well plates identified six TF null mutants with different susceptibilities to miltefosine. To validate the differential susceptibility of these mutants to miltefosine, the 6 TF null mutants were grown in the absence or presence of different miltefosine concentrations, and their radial growth was measured (Fig. 2). When compared to the wild-type strain, we observed discrete differences in five of these mutant strains (Fig. 2). Δ*mcnB* (AFUA_5G05600) strain, which encodes a homologue of A. nidulans McnB, a multicopy supressor of A. nidulans *nimA1*, (62) showed about 20% growth inhibition (Fig. 2A and B), while Δ*pacC* (AFUA_3G11970) strain, which encodes PacC, a protein important for pH regulation (63), showed about 50% inhibition compared to the wild-type strain at 8 μg/ml (Fig. 2A and B). The Δ*sslA* (AFUA_5G04333) strain, which encodes a homologue of Saccharomyces cerevisiae Ssl1p, a subunit of the general transcription factor TFIIH, has about 50% growth inhibition compared to the wild-type strain at 8 μg/ml (Fig. 2A and B). The Δ*sebA* (AFUA_4G09080) strain encodes a TF important to cope with different kinds of stress (64) and showed 40% inhibition to miltefosine at 8 μg/ml, while the wild type is inhibited 60% at this concentration (Fig. 2A and B). The AFUA_5G03030 null mutant has 65% growth inhibition compared to the wild-type strain for miltefosine at 8 μg/ml (Fig. 2). Notably, the AFUA_2G12070 mutant was unable to grow at 4 μg/ml miltefosine (Fig. 2A and B and 3A and B). AFUA_2G12070 encodes a 492-amino-acid novel fungal Zn_2_-Cys_6_ transcription factor (http://pfam.xfam.org/family/PF00172#Zn2/Cys6). We named this gene *smiA* (sensitive to miltefosine). The phylogenetic distribution of SmiA across fungal classes and genomes represents 24 species in two different taxonomic classes, Eurotiomycetes (Chaetothyriomycetidae and Eurotiomycetidae) and Sordariomycetes (Hypocreomycetidae) (Fig. 3A; see also Table S1 at https://doi.org/10.6084/m9.figshare.14762991.v4).

The Δ*smiA* strain was complemented, and the Δ*smiA*::*smiA^+^* complementing strain presented a reversible phenotype in terms of miltefosine sensitivity, indicating that the miltefosine sensitivity phenotype of the Δ*smiA* strain is due to the specific deletion of the *smiA* gene (Fig. 3B and C). The Δ*smiA* mutant has no differential susceptibility to different stress conditions, such as growth on increasing concentrations of NaCl, Calcofluor white, sorbitol, CaCl_2_, 1,4-dithiothreitol (DTT), brefeldin (growth at 44°C), and menadione (Fig. S1 at https://doi.org/10.6084/m9.figshare.14762991.v4). The wild-type and *ΔsmiA* strains have the same MICs for amphotericin, itraconazole, voriconazole, posaconazole, and caspofungin (data not shown).

Aiming to localize SmiA, we constructed a functional C-terminal SmiA-GFP strain (Fig. S2 at https://doi.org/10.6084/m9.figshare.14762991.v4) that showed no fluorescence in the absence of miltefosine (Fig. 3D and E). However, when the SmiA-GFP strain was shifted 15 min to MM supplemented with 3 μg/ml miltefosine, SmiA-GFP can be detected in about 50% of the nuclei (Fig. 3D and E). In addition, we also constructed a functional SmiA-3×HA strain (Fig. S2 at https://doi.org/10.6084/m9.figshare.14762991.v4). This strain was grown in VMM and further exposed to RPMI supplemented (or not) to an inhibitory concentration of miltefosine (12.5 μg/ml) for 4 and 8 min. A very faint band of 54.3 kDa, corresponding to SmiA-3×HA, was observed in the control not exposed to miltefosine, while increased-intensity bands were observed after 4 and 8 h of exposure to miltefosine (Fig. 3F).

These results indicate that the SmiA protein quickly translocates to the nucleus, and its expression is also increased upon miltefosine exposure.

### Miltefosine induces necrosis-like cell death and increases mitochondrial fragmentation in A. fumigatus.

**FIG 4:**
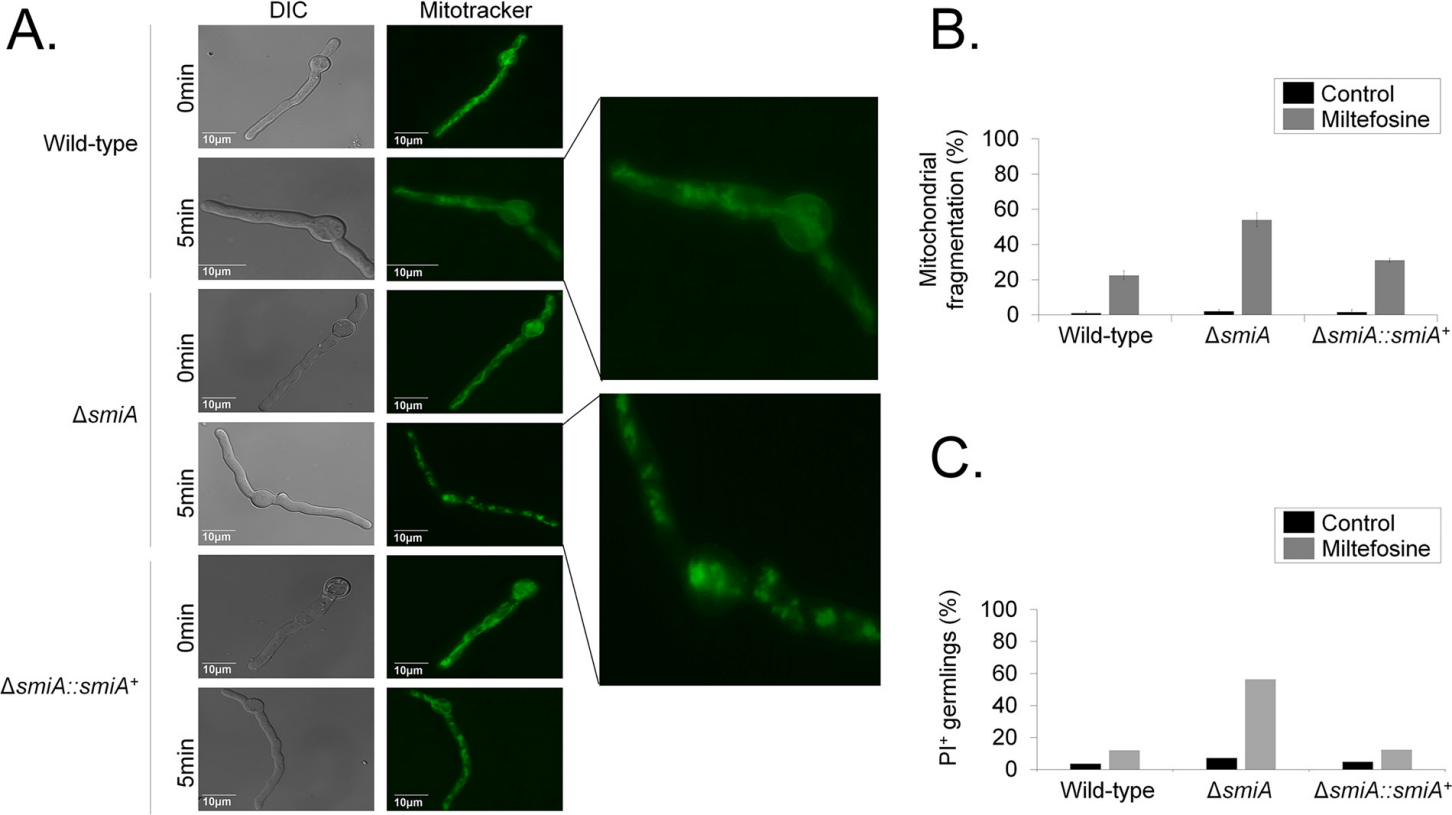
There is increased mitochondrial fragmentation and cell death when the A. fumigatus Δ*smiA* strain is exposed to miltefosine. (A) Mitochondrial morphology revealed by MitoTracker in the wild-type, Δ*smiA*, and Δ*smiA*::*smiA*^+^ strains. (B) Quantification of the mitochondrial fragmentation in the absence (control) and presence of miltefosine. (C) Quantification of PI^+^ (propidium iodide) germlings in the absence (control) and presence of miltefosine. The results are the averages ± standard deviations from three repetitions of 30 germlings for each repetition.

A. fumigatus forms mitochondrial tubular and highly dynamic networks that are fragmented in the presence of antifungal and oxidative stressing agents such as hydrogen peroxide (65, 66). This increased mitochondrial fragmentation has been described as a marker for cell death (66). Propidium iodide (PI) is a fluorescent DNA-binding dye that freely penetrates cell membranes of dead or dying cells but is excluded from viable cells. Late apoptosis and early necrosis are characterized by an increased number of PI-positive cells. To evaluate the effects of miltefosine and PI on the mitochondrial morphology and viability, germlings from the wild-type, Δ*smiA*, and Δ*smiA*::*smiA^+^* strains were treated with 3 μg/ml of the drug for 0, 5, or 10 min, and green MitoTracker (a mitochondrial fluorescent probe) or PI was added and further analyzed by fluorescence microscopy (Fig. 4A). In the absence of miltefosine, an intact mitochondrial network was observed in all three strains. However, upon 5 min of miltefosine exposure, the *ΔsmiA* cells showed about 60% mitochondrial fragmentation, evidenced by the presence of a punctated fluorescent pattern observed in the cytoplasm of the cells (Fig. 4A and B), while in the wild-type and complemented strains the levels of mitochondrial fragmentation were much lower, 20 and 30%, respectively (Fig. 4A and B). When the wild-type and the Δ*smiA*::*smiA^+^* germlings were left unexposed to miltefosine, about 5% of cells were stained by PI, while in the Δ*smiA* strain this level was about 7% (Fig. 4C). However, upon miltefosine addition, the wild-type and the Δ*smiA*::*smiA^+^* germlings were about 12% stained by PI (Fig. 4C), while more than 50% of the Δ*smiA* germlings showed PI staining (Fig. 4C). These results suggest that miltefosine induces both mitochondrial fragmentation and necrotic cell death in A. fumigatus, which was accentuated in the Δ*smiA* strain, emphasizing the importance of SmiA for survival and viability of A. fumigatus.

**FIG 5:**
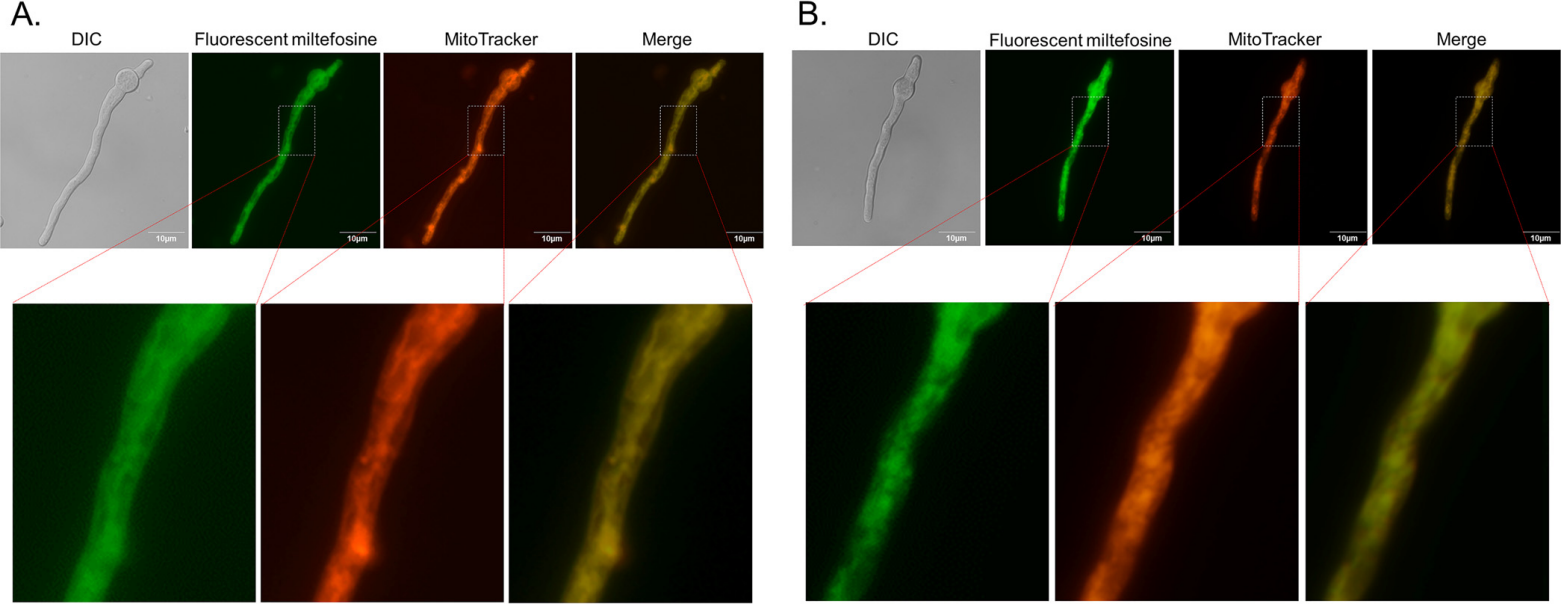
Fluorescent miltefosine analogue MT-11C-BDP is localized in the mitochondria. (A and B) A. fumigatus germlings (16 h of growth in MM) were exposed for 5 min to 4 μg/ml MT-11C-BDP. Germlings were stained with MitoTracker Deep Red FM.

A. fumigatus germlings were exposed to 4 μg/ml a functional fluorescent analogue of miltefosine, MT-11C-BDP [11-(4,4-difluoro-1,3,5,7-tetrametil-4-bora-3a,4a-diaza-s-indacen-2-il) n-undecilfosfatidilcolina] (67), for about 5 min (Fig. 5). MT-11C-BDP localizes to tubular structures that resemble mitochondrial networks and were also fragmented in a fraction of the germlings (Fig. 5A and B). Colocalization with MitoTracker Deep Red FM indicated that the MT-11C-BDP analogue is mainly localized at the mitochondria.

### Miltefosine induces the modulation of genes encoding proteins responsible for the metabolism of lipids, fatty acids, and derivatives.

Aiming to get insights about genes that are modulated under miltefosine exposure, we carried out a transcriptomic analysis (RNA-seq) analyzing the A. fumigatus wild-type strain exposed to miltefosine. Compared to the wild type grown in MM, when the cells were shifted to RPMI medium supplemented with 3 μg/ml miltefosine for 30 min, a total of 1,248 genes were upregulated (log_2_ fold change [log_2_FC] > 1.0; *P *< 0.005), and 940 genes were downregulated (log_2_FC < −1.0; *P* < 0.005). In both cases the false discovery rate (FDR) was less than 0.05 (Table S2 at https://doi.org/10.6084/m9.figshare.14762991.v4).

**FIG 6:**
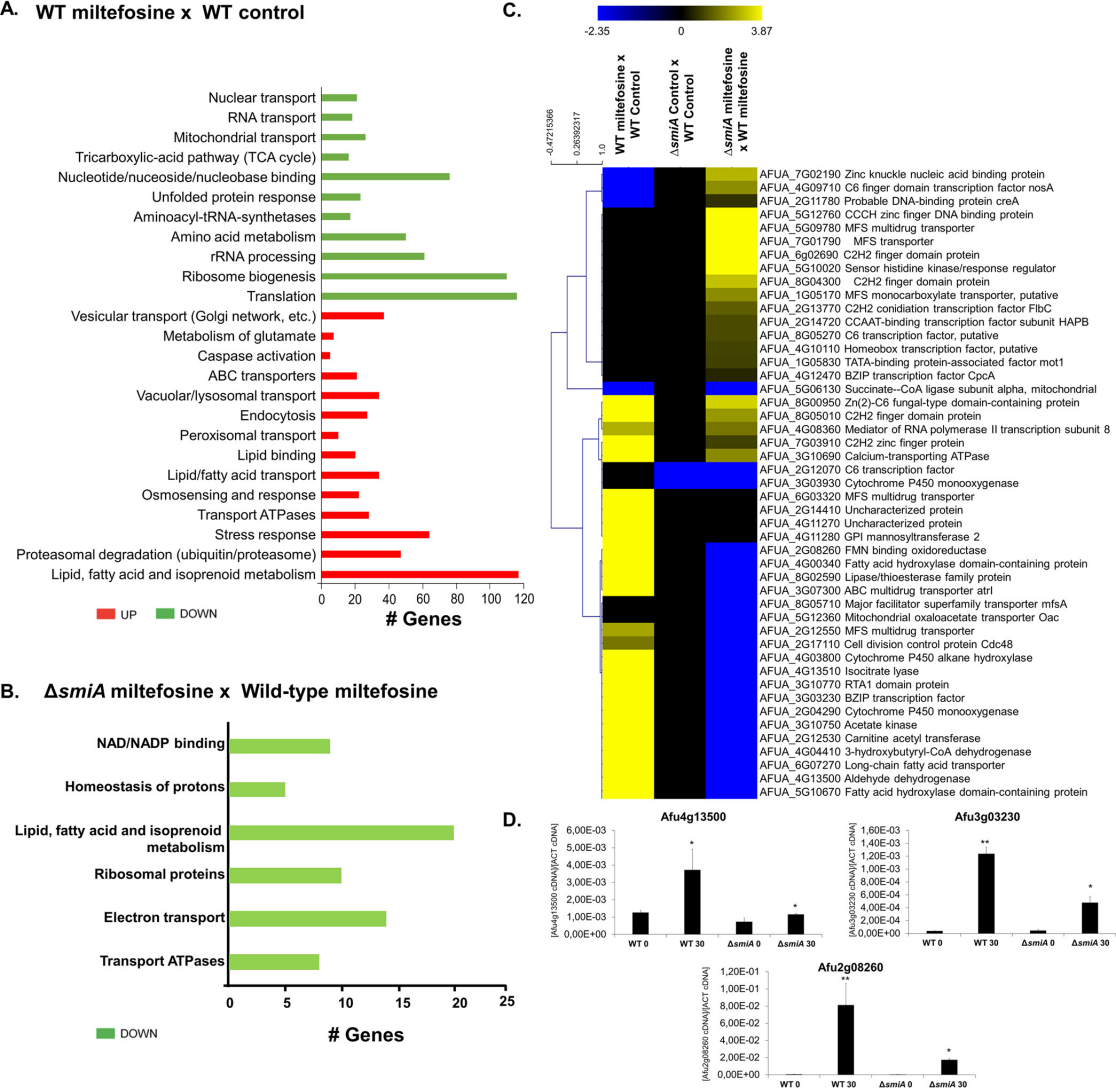
Transcriptional profiling of A. fumigatus wild-type and *ΔsmiA* strains exposed to miltefosine. (A) FunCat categorization of differently expressed genes (DEGs) up- and downregulated in the wild-type strain exposed to miltefosine compared to the wild-type strain grown in VMM (control). (B) FunCat analysis of DEGs downregulated in the Δ*smiA* strain under miltefosine exposure compared to the Δ*smiA* strain grown in VMM. (C) Heat map of log_2_ fold change (log_2_FC) of DEGs as determined by RNA-seq. Log_2_FC values are based on comparisons between (i) wild-type strain exposed to miltefosine versus wild-type strain in grown in MM (control); (ii) Δ*smiA* strain grown in VMM (control) versus wild type grown in VMM (control); and (iii) Δ*smiA* strain exposed to miltefosine versus wild-type strain under miltefosine treatment. Hierarchical clustering was performed in Multiple Experiment Viewer (MeV) (http://mev.tm4.org/), using Pearson correlation with complete linkage clustering. Heat map scale and gene identities are shown. (D) Validation of RNA-seq data. Expression of three genes as determined by qRT-PCR after 0 and 30 min of exposure to 3 μg/ml miltefosine. Gene expression values were normalized by the expression of β-tubulin. Standard deviations are shown for biological triplicates.

The enrichment analysis using FunCat (https://elbe.hki-jena.de/fungifun/fungifun.php) showed a transcriptional upregulation of genes involved in vesicular and vacuolar transport, metabolism of glutamate, caspase activation, ABC transporters, osmosensing response, transport ATPases, stress response, proteasomal degradation, lipid transport, and high enrichment in lipid, fatty acid, and isoprenoid metabolism (Fig. 6A). Genes involved in nuclear transport, RNA transport, mitochondrial transport, tricarboxylic acid (TCA) cycle, nucleotide binding, unfolded protein response, aminoacyl-tRNA-synthetases, amino acid metabolism, rRNA processing, ribosome biogenesis, and translation were downregulated upon miltefosine exposure (Fig. 6A). These results suggest that under miltefosine treatment, A. fumigatus increases the expression of genes involved in fatty acid metabolism and transport, stress responses, and specific transporters, while it represses mitochondrial functions (e.g., TCA cycle and mitochondrial transport) and amino acid and protein biosynthesis.

### *smiA* is important for the induction of genes involved in lipid metabolism upon miltefosine exposure in A. fumigatus.

To identify potential targets modulated by SmiA, we performed transcriptional profiling of the Δ*smiA* null mutant under the same experimental design described for the wild-type strain. We identified 292 differentially expressed genes (DEGs), with 184 genes upregulated (log_2_FC, >1.0; *P *< 0.005) and 108 genes downregulated (log_2_FC, <−1.0; *P* < 0.005; FDR < 0.05) upon miltefosine exposure (Table S3 at https://doi.org/10.6084/m9.figshare.14762991.v4). FunCat enrichment analysis has not shown categories for upregulated genes in the Δ*smiA* mutant. However, FunCat for the downregulated genes in the Δ*smiA* mutant exposed to miltefosine revealed enrichment for categories of genes encoding proteins involved in lipid, fatty acid, and isoprenoid metabolism, NAD/NADP binding, homeostasis of protons, ribosomal proteins, electron transport, and transport of ATPases (Fig. 6B).

A visual inspection of DEGs in both wild-type and Δ*smiA* strains showed zinc finger proteins (AFUA_5G12760 and AFUA_6g02690), MFS transporters (AFUA_5G09780 and AFUA_7G01790), and a sensor histidine kinase regulator (AFUA_5G10020) with higher levels of expression in the Δ*smiA* strain than the wild type (Fig. 6C). Genes involved in the metabolism of fatty acids (AFUA_4G00340, AFUA_2G12530, AFUA_6G07270, AFUA_5G10670, and AFUA_8g02590), cytochrome P450 enzymes (AFUA_3g03930, AFUA_4G03800, and AFUA_2G04290), cell division control protein (AFUA_2G17110), aldehyde dehydrogenase (AFUA_4G13500), and isocitrate lyase (AFUA_4G13510) were downregulated in the Δ*smiA* strain compared to the wild-type strain (Fig. 6C). Accordingly, the RNA-seq data were validated by performing real-time PCR on 3 selected genes that showed a very similar expression pattern compared with data from RNA-seq (Fig. 6D).

Taken together, our data show that in the wild-type strain, lipid and fatty acid metabolism are upregulated upon miltefosine exposure, suggesting their importance for survival in the presence of this drug. On the other hand, the deletion of the *smiA* gene leads to a deficiency in the lipid and fatty acid metabolism, strongly suggesting that it is linked to the higher sensitivity of this mutant to miltefosine.

### SmiA binds to a discrete number of gene promoter regions specifically in the presence of miltefosine.

**FIG 7:**
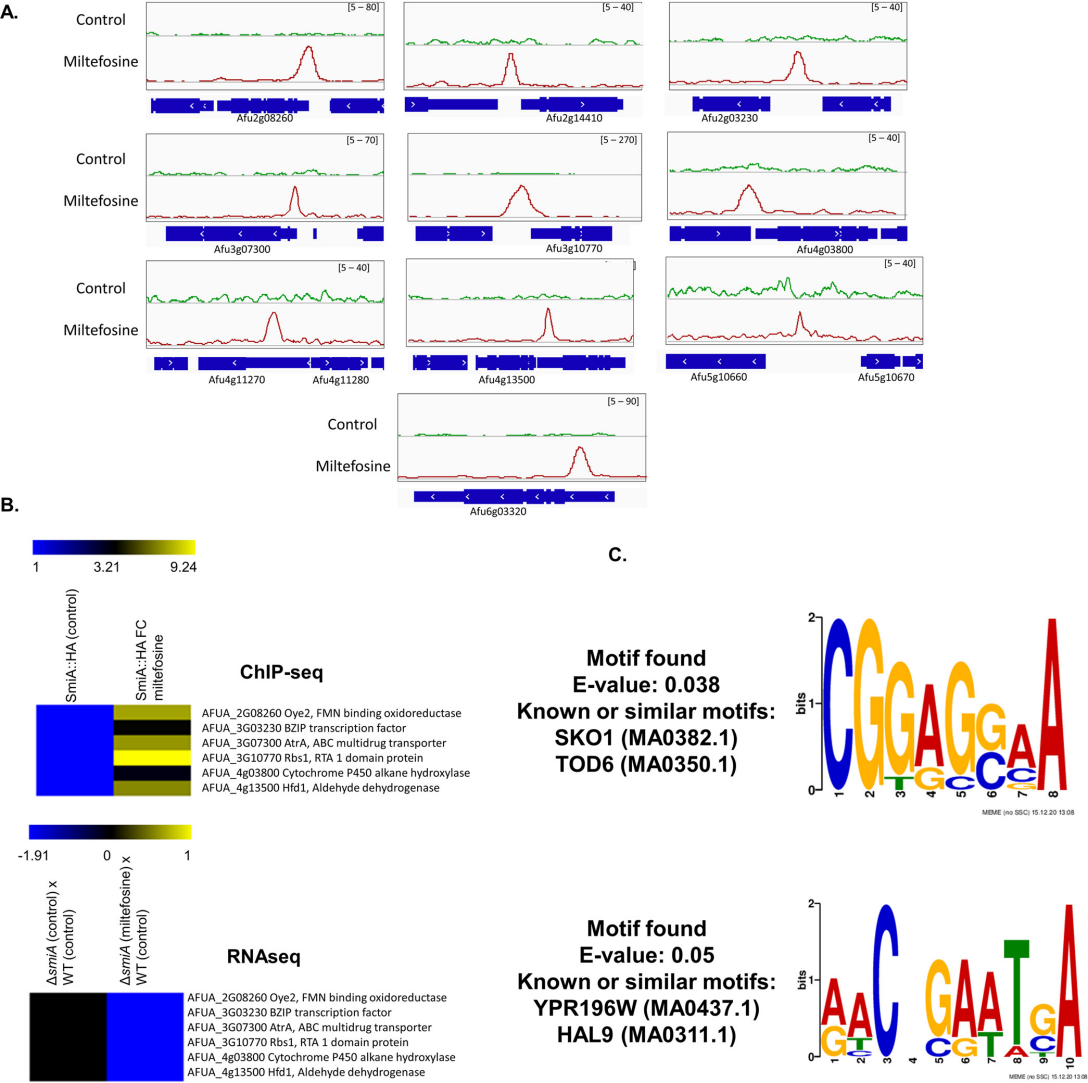
ChIP-seq of the SmiA-3×HA strain exposed or not to miltefosine. (A) ChIP-Seq Integrative Genomics Viewer (IGV; http://software.broadinstitute.org/software/igv/download) screenshot for promoter regions of genes that bound to SmiA-3×HA when grown for 24 h in VMM or after the addition of 12.5 μg/ml miltefosine for 30 min. (B) Heat map of the ChIP-seq results for 6 genes showing the fold enrichment of SmiA binding after 0 and 30 min of exposure to 12.5 μg/ml miltefosine and the RNA-seq results for the same 6 genes in the WT and Δ*smiA* strains after exposure to 3 μg/ml during 30 min. (C) MEME-ChIP analysis of the 500-bp region surrounding the peaks identified in the ChIP-seq analysis.

Considering that SmiA seems to be a TF involved in miltefosine resistance in A. fumigatus, we decided to identify potential direct targets under SmiA control of this protein using the ChIP-seq approach. The SmiA-3×HA strain (Fig. 7A and Fig. S2 at https://doi.org/10.6084/m9.figshare.14762991.v4) was grown in MM and further exposed to RPMI supplemented (or not) with miltefosine for 30 min. After immune precipitation using anti-HA antibody, samples were sequenced using the Illumina HiSeq2500 platform, the reads were aligned to the A. fumigatus Af293 reference strain, and the program MACS2 was used for peak calling. The peak intensity map showed that SmiA binding was enriched at the promoter region of 12 specific genes that are present in different chromosomes: (i) AFUA_2G08260, encoding a homologue of S. cerevisiae Oye2p, an NADPH oxidoreductase containing flavin mononucleotide (FMN) that may be involved in sterol metabolism, oxidative stress response, and programmed cell death (www.yeastgenome.org); (ii) AFUA_3G03230, encoding a BZIP transcription factor (www.fungidb.org); (iii) AFUA_3G07300, AtrA encoding an ABC multidrug transporter (www.fungidb.org); (iv) AFUA_3G10770, encoding a homologue of S. cerevisiae Rbs1p, a sphingoid long-chain base (LCB) efflux transporter, integral membrane transporter that localizes to the plasma membrane and may transport LCBs from the cytoplasmic side toward the extracytoplasmic side of the membrane, and a role in glycerophospholipid translocation (www.yeastgenome.org); (v) AFUA_4G03800, encoding a cytochrome P450 alkane hydroxylase (www.fungidb.org); (vi) AFUA_4G13500, encoding a homologue of S. cerevisiae Hfd1p, a dehydrogenase involved in ubiquinone and sphingolipid metabolism, converting hexadecenal to hexadecenoic acid in sphingosine 1-phosphate catabolism, the human homologue of ALDH3A2, mutated in Sjogren-Larsson syndrome (www.yeastgenome.org) (68); (vii) AFUA_5G10670, encoding a protein that has a domain(s) with predicted iron ion binding, oxidoreductase activity, and role in fatty acid biosynthetic process, oxidation-reduction process (www.fungidb.org); (viii) AFUA_2G14410, encoding an orthologue that has a role in xanthophyll metabolic processes (www.fungidb.org); (ix) AFUA_4G11270, encoding an unknown function hypothetical protein (www.fungidb.org); (x) AFUA_4G11280, encoding an orthologue that has dolichyl-phosphate-mannose-glycolipid alpha-mannosyltransferase activity and role in the glycophosphatidylinositol (GPI) anchor biosynthetic process (www.fungidb.org); (xi) AFUA_5G10660, encoding a pentatricopeptide repeat protein (www.fungidb.org); and (xii) AFUA_6G03320, encoding an MFS transporter (www.fungidb.org) (Fig. 7A and Table S4 at https://doi.org/10.6084/m9.figshare.14762991.v4). SmiA binding to these promoter regions happens specifically in the presence of miltefosine, suggesting that SmiA is important for the activity of those genes in the presence of this drug. Accordingly, the RNA-seq data demonstrate that the expression levels of AFUA_2G08260, AFUA_3G03230, AFUA_3G07300, AFUA_3G10770, AFUA_4G03800, AFUA_4G13500, and AFUA_5G10670 are repressed in the Δ*smiA* strain compared with the wild type when both strains are exposed to miltefosine (Fig. 7B and Table S4 at https://doi.org/10.6084/m9.figshare.14762991.v4).

To identify putative SmiA-binding motifs in A. fumigatus, we carried out multiple expectation maximum for motif elicitation (MEME) of the 500 nucleotides surrounding each peak sequence identified in the ChIP-seq. The results show the enrichment of two consensus DNA binding sequences for SmiA in the presence of miltefosine (Fig. 7C). Two binding motifs were predicted, 5′-CGGAG(G or C)AA-3′ (E value of 5e−02), and 5′-AACNGAATGA-3′ (E value of 3.8e−02).

Together, our data highlight the importance of SmiA for events involved with the miltefosine resistance process in A. fumigatus and suggest that genes potentially modulated by the SmiA binding have specific binding motifs for this protein. Several promoter regions of genes that are bound by SmiA encode proteins involved in lipid metabolism.

### SmiA is important for sphingolipid biosynthesis.

**FIG 8:**
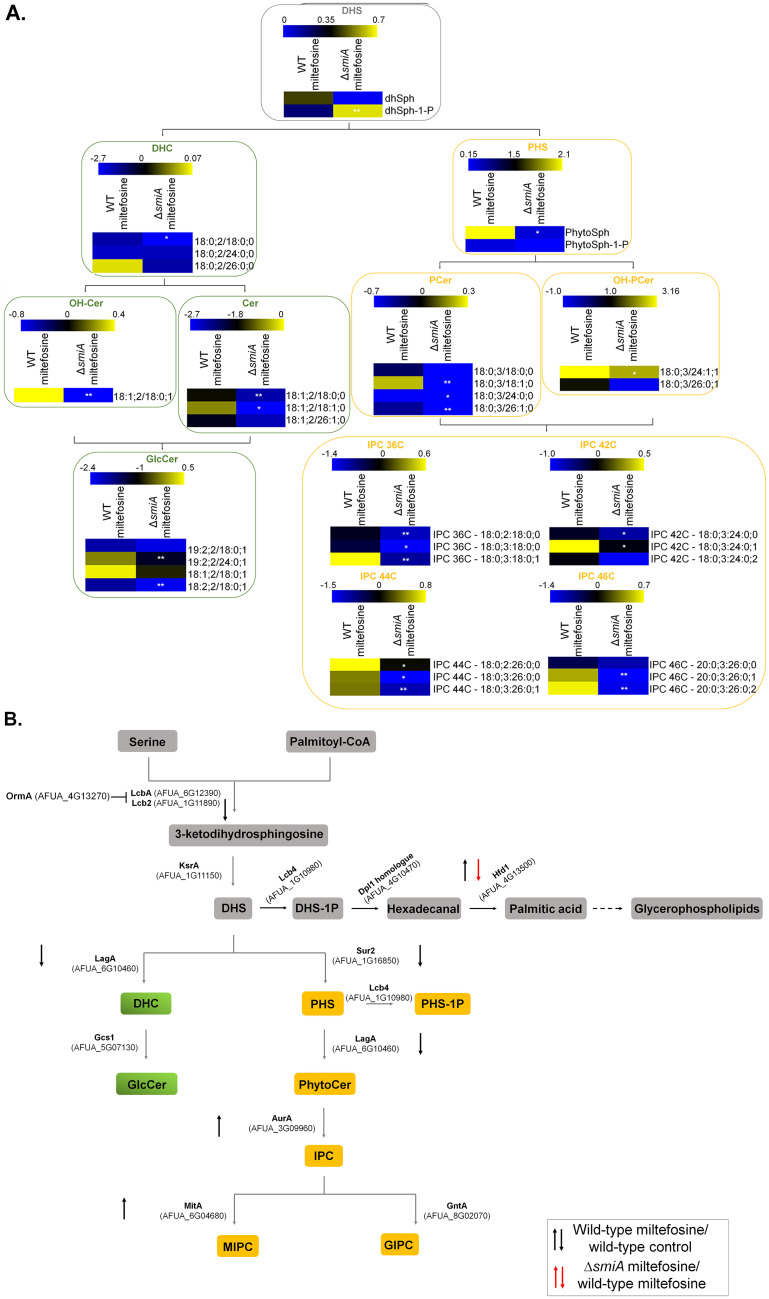
Deletion of *smiA* leads to an overall reduction of sphingolipids biosynthesis in A. fumigatus. (A) The wild-type and Δ*smiA* strains were grown in liquid VMM for 16 h and transferred to RPMI medium supplemented (or not) with 3 μg/ml miltefosine for an additional 4 h, and the sphingolipids were extracted and measured by mass spectrometry. Heat map labels surrounded by boxes with gray borders represent the intermediates of the SL biosynthetic pathway. Heat maps surrounded by boxes with green borders represent the intermediates from the neutral branching, while heat maps surrounded by boxes with yellow borders represent the intermediates from the acidic branch of the SL biosynthetic pathway. Heat maps show the values obtained by the VMM/RPMI ratio. Experiments were performed by using three independent biological experiments, and the results are averages of them. Statistical analysis was performed using Student's *t* test (*P* < 0.05). (B) Diagram showing the different genes involved in sphingolipid biosynthesis. Expression levels of genes encoding enzymes involved in the sphingolipids biosynthesis were selected from the RNA-seq analysis. DHS, dihydrosphingosine; DHS-1P, dihydrosphingosine 1-phosphate; DHC, dihydroceramide; Cer, ceramide; GlcCer, glucosylceramide; OH-Cer, hydroxy-ceramide; PHS, phytosphingosine; PHS-1P, phytosphingosine 1-phosphate; PCer, phytoceramide; OH-PCer, hydroxy-phytoceramide; IPC, inositolphosphoryl ceramide.

Our previous results suggest that myriocin, a sphingolipid inhibitor, impairs the antifungal activity of miltefosine (Fig. 1F). Considering that the metabolism of lipids seems to be involved with miltefosine resistance and the TF SmiA is linked to this process, we performed the SL profiling of both wild-type and Δ*smiA* strains exposed to miltefosine. Both strains were grown in VMM for 16 h and shifted to RPMI medium supplemented (or not) with 3 μg/ml miltefosine for 4 h. The main SL intermediates starting from the branching point of the pathway (dihydrosphingosine [DHS]) were then measured through mass spectrometry analysis, and the results were expressed as fold increase or decrease compared to the control not exposed to miltefosine (Fig. 8A).

The deletion of *smiA* leads to an overall reduction of the analyzed SLs compared to the SL levels in the wild-type strain under the same conditions (Fig. 8A). Interestingly, the reduction in SL levels occurs in both acidic and neutral branches of the pathway, suggesting the deletion of *smiA* affects the early steps of the SL biosynthetic process. The branching point of the SL pathway is DHS, the precursor of dihydroceramide (DHC; the first intermediary of the neutral branch) and phytosphingosine (PHS; the first intermediary of the acidic branch). However, DHS is also converted to dihydrosphingosine 1-phosphate (DHS-1P), starting the metabolic pathway where the DHS-1P is converted to glycerolipid through many enzymatic reactions (Fig. 8A). Upon miltefosine exposure, there is an increase of DHS and a decrease in DHS-1P in the wild-type strain, while the opposite is observed in the Δ*smiA* mutant (Fig. 8A). PHS, ceramide (CER), hydroxyceramide (OH-CER), phosphoceramide (P-CER), hydroxyphosphoceramide (OH-PCER), glucoceramide (GLC-CER), and inositolphosphoryl-ceramide (IPC) are increased when the wild-type strain is exposed to miltefosine (Fig. 8A). In contrast, all these sphingolipids were reduced in the Δ*smiA* strain when exposed to miltefosine (Fig. 8A). We investigated in our RNA-seq data set the expression levels of the genes that encode enzymes involved in the different steps of the SL pathway (Fig. 8B). We observed 4 genes (Lcb2, AFUA_1G11890; KsrA, AFUA_1G11150; LagA, AFUA_6G10460; and Sur2, AFUA_1G16850) with reduced and 2 genes (AurA, AFUA_3G09960; MitA, AFUA_6G04680) with increased expression when the wild type was exposed to miltefosine (Fig. 8B). Interestingly, only one gene (Hfd1, AFUA_4G13500) is differentially expressed with reduced expression when the Δ*smiA* mutant is exposed to miltefosine (Fig. 8B). Taken together, our results suggest that miltefosine antifungal activity against A. fumigatus interferes directly in the SL biosynthesis pathway.

### Azole-resistant clinical isolates of A. fumigatus are sensitive to miltefosine.

To verify if miltefosine is a good candidate for therapy against azole-resistant strains, we tested if miltefosine could inhibit A. fumigatus growth of 19 clinical isolates (in addition to CEA17 strain) with different levels of azole resistance by determining their MICs. We tested 9 azole-sensitive A. fumigatus strains (CEA17, CYP15-109, IF1S-F4, IFM59056, ISFT-021, IFM61407, MO68507, MO54056, and IFM59056) and 10 azole-resistant isolates with different resistance mechanisms, cultured from different sample sites from patients from Portugal, Japan, Belgium, and Switzerland (69) (Table 2). The most common azole resistance mechanisms include amino acid substitutions in the target Cyp51A protein and tandem repeat sequence insertions at the *cyp51A* promoter (70). The *cyp51A* gene is not mutated in the azole-resistant strains F16134, F14946, CYP15-117, CYP15-147, CYP15-75, CYP15-93, CYP15-106, and CYP15-115 (*cyp51A* was not sequenced in the CYP-15-91 strain), suggesting different mechanisms of azole resistance (69). In contrast, strains 1799392 and 20089320 have TR34 tandem repeats at the *cyp51A* promoter region and L98H amino acid replacement at Cyp51A (71). All the azole-sensitive or -resistant clinical isolates have a MIC of 4 μg/ml miltefosine (Table 2). These results strongly indicate that miltefosine can inhibit the growth of clinical isolates that have developed resistance to azoles through different mechanisms.

**TABLE 2:** MIC of A. fumigatus clinical isolates in the presence of different antifungal drugs

Strains	MIC (μg/μl)				
	Itraconazole	Posaconazole	Voriconazole	Amphotericin B	Miltefosine
CEA17	2	2	1	0.5	4
F16134	>8	>8	4	0.25	4
F14946	>8	8	8	0.25	4
CYP15-117	>8	2	2	0.5	4
CYP15-147	>8	2	8	1	4
20089320	>8	4	4	1	4
CYP15-75	8	4	8	0.5	4
CYP15-91	8	1	2	1	4
CYP15-93	8	1	2	0.5	4
CYP15-106	8	2	1	1	4
CYP15-115	8	0.5	0.5	0.25	4
17993925	8	2	4	1	4
CYP15-109	4	1	>8	0.5	4
IF1S-F4	4	1	1	0.5	4
IFM59056	4	1	0.125	0.5	4
ISFT-021	2	1	0.25	1	4
IFM61407	2	2	0.125	0.5	4
MO68507	1	1	0.25	0.5	4
MO54056	1	1	0.125	0.5	4
IFM59056	1	1	0.125	0.5	4

We also observed that several A. nidulans, A. niger, and A. lentulus clinical and environmental isolates have MICs of 4 μg/ml, while an environmental isolate of A. flavus has a MIC of 8 μg/ml (Table S5 at https://doi.org/10.6084/m9.figshare.14762991.v4).

### Miltefosine increases the survival of Galleria mellonella larvae infected with A. fumigatus.

**FIG 9:**
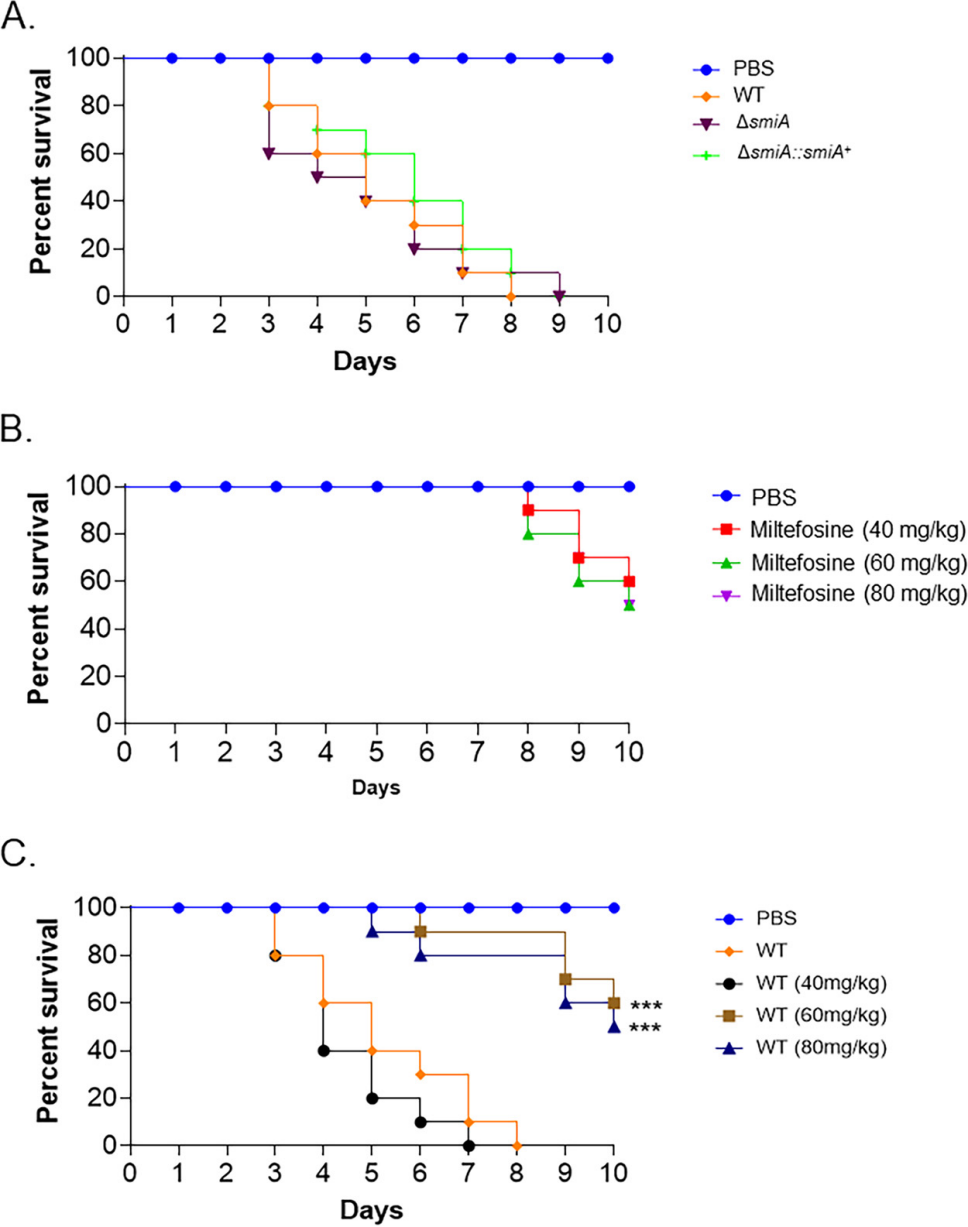
Miltefosine contributes to reduce A. fumigatus virulence in G. mellonella wax moth. (A to C) Survival curves of G. mellonella larvae (*n* = 10/strain) infected via injection with 10^6^ conidia from wild-type, Δ*smiA*, and Δ*smiA*::*smiA^+^* strains. Larvae were monitored for a period of 10 days postinfection. Log rank (Mantel-Cox) tests (***, *P < *0.001) were used to compare all the treatments with the larvae infected with the A. fumigatus wild-type control.

On the basis of its essential role in sphingolipid biosynthesis, we asked whether SmiA is important for A. fumigatus virulence. G. mellonella larvae (*n* = 10 for each strain) were infected with the wild-type, *smiA* deletion, and complementation strains, and survival was assessed over a time period of 10 days (Fig. 9A). The wild‐type, Δ*smiA*, and Δ*smiA*::*smiA^+^* strains caused 90% to 100% mortality after 9 to 10 days postinfection (p.i.) (*P* < 0.001) (Fig. 9A). These results indicate that SmiA is not a key regulator of A. fumigatus pathogenesis in the G. mellonella model.

As a proof of principle of the *in vivo* antifungal activity of miltefosine, we tested its ability to control or reduce the A. fumigatus infection in G. mellonella larvae (Fig. 9B and C). First, we tested three different concentrations of miltefosine (40, 60, and 80 mg/kg of the body weight of the larva), aiming to verify the drug concentration that could cause minimal damage to the larvae. These miltefosine concentrations caused about 40% mortality after 10 days (*P* < 0.001) (Fig. 9B). A. fumigatus infection of G. mellonella larvae combined with 60 and 80 mg of miltefosine/kg of larva resulted in about 50% survival of the invertebrate host (*P* < 0.001) (Fig. 9C). These results indicate that miltefosine is able to control 50% of the mortality caused by A. fumigatus infection in G. mellonella.

## DISCUSSION

In recent years, the incidence of fungal infections has grown dramatically, leading to an increasing number of deaths worldwide (1, 2). The mortality rate is linked to a set of conditions, such as host immune system integrity, availability of an effective antifungal drug, and the occurrence of clinical resistant isolates (6, 7, 11, 21, 72). Invasive pulmonary aspergillosis (IPA) is a disease caused by the opportunistic human pathogen A. fumigatus and displays high levels of morbidity and mortality mainly in immunocompromised patients (1, 17). Azoles are the main drug used to control IPA, but the azole-resistant A. fumigatus isolates have increased significantly over the last decade (28, 33–38).

Given this scenario, there is an urgent need for new antifungal therapies applied to control IPA and other fungal diseases. The development of new antifungal drugs raises challenges, such as the high costs and the time required for development and licensing of new compounds. To circumvent the slowness and cost of developing new drugs, the screening of chemical libraries and repurposing of drugs that are already commercialized for other purposes is a great opportunity to discover new antifungal compounds (43, 45, 48, 52, 73–75). Here, we screened the growth of A. fumigatus in the presence of compounds present in two drug libraries and identified 10 compounds, among them five compounds already known as inhibitors of fungal growth, including two azole derivatives (econazole nitrate and oxiconazole nitrate), fluvastatin, which inhibits ergosterol biosynthesis, and iodoquinol and miltefosine, drugs with an unknown mechanism of action. To our knowledge, the other five identified compounds (mesoridazine, cisapride, indinavir sulfate, enalaprilat, and vincristine sulfate) are novel as antifungal agents and have not been reported before. We investigated a possible mechanism of action for miltefosine, a chemical belonging to the alkylphosphocholine class. Miltefosine is mainly localized in the mitochondria and has a MIC of 4 μg/ml under *in vitro* conditions, and we demonstrated that miltefosine is able to inhibit, to the same extent, A. fumigatus growth of several clinical isolates, including highly azole-resistant strains. Miltefosine was a drug initially used as an antineoplastic drug (76) and for treatment against trypanosomatids (77), and it is the first drug approved for oral treatment of leishmaniasis (58). However, the mechanism of action of miltefosine is not fully understood and not necessarily the same in different organisms, and the specific target of miltefosine has not been identified yet. Recent studies in trypanosomatids have suggested that miltefosine acts by (i) altering the correct functionality of the sterol and sphingolipid metabolism (58, 59); (ii) inhibiting the phosphatidylcholine synthesis (78) and membrane remodeling due to the phospholipase action, contributing to changing membrane physical properties (79); (iii) inhibiting cytochrome *c* oxidase (80); (iv) activating the plasma membrane Ca^+2^ channel opened by the sphingolipid sphingosine; and (v) destabilizing the intracellular Ca^+2^ homeostasis (59). On the other hand, the resistance phenotype to miltefosine in trypanosomatids has been linked to genes belonging to lipid metabolism (81).

Concerning its antifungal behavior, miltefosine has been demonstrated to be effective against different fungal species (82–93); however, its mode of action remains to be clarified. Recent studies have suggested that miltefosine triggers its antifungal effects by destabilizing cell membranes and inducing apoptosis (53, 84, 87, 92, 94). Accordingly, Spadari and colleagues demonstrated that for Cryptococcus spp., miltefosine affects the plasma membrane permeability due to its interaction with ergosterol and/or phospholipids, increasing the production of reactive oxygen species and DNA fragmentation, which culminates in fungal death by apoptosis (87). In addition, in C. krusei, the mode of action of miltefosine is also supposed to be related to the binding of the drug to ergosterol in the cell membrane, leading to cell apoptosis (92).

We observed that at the MIC, miltefosine displayed a fungicidal effect against A. fumigatus, corroborating previous results presented for several fungal species, such as Cryptococcus spp., *Candida* spp., and molds (83–88, 90–92, 95). Our studies showed that miltefosine could decrease A. fumigatus mortality 50% in G. mellonella larvae. We then decided to check if miltefosine could present any interaction with other antifungal drugs. Azoles such as posaconazole and voriconazole, which act by inhibiting the ergosterol biosynthesis (96), amphotericin B, which sequesters ergosterol from the cell membrane (97), and caspofungin, which targets the glucan synthase Fks1 and inhibits the synthesis of β-(1,3)glucan (98), were included in our analysis, and none of them showed interaction with miltefosine. Our results corroborated what was previously observed in Aspergillus spp. where the interaction between these compounds with miltefosine was indifferent for 32 from 33 isolates (85). In contrast to our results, miltefosine has been reported to have synergy with posaconazole against Fusarium oxysporum and the mucormycetes (99). In addition, a recent study with C. auris demonstrated that for 25% of the isolates assessed, there was a synergic activity between miltefosine and amphotericin B, with an FICI of 0.5 (92).

Sphingolipids are complex lipids composed of octadecacarbon alkaline blocks, synthesized from nonsphingolipid precursors, and represent one of the most abundant lipids in eukaryotic cell membranes (100, 101). In fungi, SLs are involved in central cellular functions, such as growth, pathogenesis, cell death, and signal transduction (102–104). SL biosynthesis starts in the endoplasmic reticulum, where the nonlipidic precursors serine and palmitoyl coenzyme A are condensed by the serine palmitoyltransferase enzyme (SPT) into 3-keto dihydrosphingosine. The SPT is specifically targeted by myriocin, a sphingolipid inhibitor (60). The interaction assay between miltefosine and myriocin showed that at high concentrations of both compounds, the FICI value was greater than 4.0, characterizing an antagonistic effect between these drugs (57). This indicates that sphingolipid metabolism may be important to the antifungal effect of miltefosine, corroborating previous results obtained for other fungal species and trypanosomatids (58, 59, 87, 92).

We were able to identify a completely novel A. fumigatus transcription factor, SmiA, linked to miltefosine resistance in this pathogen. This information came from a large-sale phenotypic screening of a collection of TF deletion mutants in the presence of miltefosine. Although the deletion of six TFs somehow moderately impacted the growth of the mutant in the presence of miltefosine, the Δ*smiA* mutant is the most sensitive mutant. SmiA is a novel and uncharacterized TF that codifies a putative Zn(II)Cys6 binuclear domain that translocates to the nucleus in the presence of miltefosine and seems to be a key TF in the miltefosine response in A. fumigatus. Miltefosine at MIC completely abolished Δ*smiA* mutant growth, and no additional phenotypes were observed under other stress conditions, such as growth in the presence of subinhibitory concentrations of posaconazole, voriconazole, caspofungin, NaCl, Calcofluor white, sorbitol, and CaCl_2_. The identification of the *smiA* gene as a putative major TF involved in A. fumigatus response to miltefosine provided us with an opportunity to inquire into the molecular mechanisms that are regulated by this gene.

The transcriptional profiling through RNA-seq assay with the wild-type strain in the presence or absence of miltefosine indicated increased upregulation of genes involved in lipids/fatty acid transport and metabolism. In contrast, the RNA-seq of the Δ*smiA* mutant exposed to miltefosine shows exactly the opposite behavior. Lipid and fatty acid metabolism was the main category of downregulated genes, which strongly suggests that this TF participates directly or indirectly in the induction of genes involved in lipid metabolism, including genes involved in the biosynthesis of sphingolipids. The identification of SmiA represents the first genetic element described and characterized that plays a direct role in miltefosine response in fungi.

Our work provides opportunities for understanding the mechanism of action of miltefosine through the characterization of the genes that are differentially expressed in the Δ*smiA* mutant. Further work will focus on the molecular characterization of these differentially expressed genes.

## MATERIALS AND METHODS

### Media, strains, and phenotypic characterization.

The Aspergillus spp. used in this work are listed in Table S5 at https://doi.org/10.6084/m9.figshare.14762991.v4. All Aspergillus strains were grown in either solid minimal medium (MM; 1% [wt/vol] glucose, 50 ml of a 20× salt solution, trace elements, 2% [wt/vol] agar, pH 6.5) or solid complete medium (YAG; 2% [wt/vol] glucose, 0.5%[wt/vol] yeast extract, trace elements, 2% [wt/vol] agar) at 37°C. The composition of the trace elements and nitrate salts is described by Käfer (105). For RNA-seq, ChIP-seq, and lipidomics, conidia were germinated in RPMI 1640 media and transferred to liquid Vogel’s minimal medium (VMM). For phenotypic characterization, plates containing solid MM were centrally inoculated with 10^5^ spores of each strain in the presence or absence of various concentrations of miltefosine (0 to 8 μg/ml). After 120 h of incubation at 37°C, radial growth was measured. All plates were grown in triplicate, and averages ± standard deviations (SD) of the data are plotted. All strains used in this work are listed in Table S6 at https://doi.org/10.6084/m9.figshare.14762991.v4.

### Library drug screenings.

Two different drug libraries were screened for antifungal activity against A. fumigatus CEA17 strain, the Pathogen Box (https://www.mmv.org/mmv-open/pathogen-box) and the National Institutes of Health (NIH) clinical collection (NCC) (https://pubchem.ncbi.nlm.nih.gov/source/NIH%20Clinical%20Collection). The Pathogen Box (https://www.mmv.org/) is a collection of 400 diverse, drug-like molecules with already-described activity against different pathogens responsible for important neglected diseases, such as malaria, tuberculosis, toxoplasmosis, and others. The NCC library is composed of a small-molecule repository of 727 compounds, which are part of the screening library for the NIH Roadmap Molecular Libraries Screening Centers Network (MLSCN), corresponding to a collection of chemically diverse compounds that have been in phase I to III clinical trials (45).

For the primary screening, the drugs were diluted from 0.78 to 25 μM in 200 μl of MOPS [3-(N-morpholino) propanesulfonic acid)-buffered RPMI 1640 (Life Technologies), pH 7, in 96-well plates. In each well, a total of 1 × 10^4^ conidia of A. fumigatus wild-type strain was inoculated. Plates were incubated for 48 h at 37°C without shaking. Wells containing only medium and dimethyl sulfoxide (DMSO) were used as controls. Fungal growth inhibition was determined visually as a no-growth endpoint, and those compounds were selected for further studies. All experiments were done in triplicate.

Fungicidal or fungistatic activity of the selected compounds was also assessed. Briefly, a total of 1 × 10^4^ conidia of A. fumigatus wild-type strain was inoculated in 96-wells plates, each well containing 200 μl of MOPS-buffered RMPI 1640 medium plus the lowest concentration of each compound that promoted fungal growth inhibition in the primary screening. Plates were incubated for 48 h at 37°C without shaking. Following, 100 conidia were plated in solid complete medium and incubated at 37°C for another 36 h. Wells containing only medium and DMSO were used as controls. The number of viable colonies was determined by CFU number compared to the negative control (no drug), which had 100% survival. Results are expressed as means and standard deviations (SD) from three independent experiments.

### MIC.

The miltefosine drug used for MIC assays was purchased from Sigma-Aldrich and solubilized in ethanol. The MIC was determined based on the M38-A2 protocol of the Clinical and Laboratory Standards Institute (106).

Briefly, the assay was performed in 96-well plates containing 200 μl of MOPS-buffered RPMI 1640 medium, pH 7.0, supplemented with miltefosine (0 to 8 μg/ml] and 1 × 10^4^ conidia of A. fumigatus per well. Plates were incubated at 37°C without shaking for 48 h. Wells containing only medium and ethanol were used as a control. The MIC was defined as the lowest concentration of miltefosine that visually inhibited 100% of fungal growth. All experiments were done in triplicate.

### Assays for checking antifungal activity of drug combinations.

We checked the interaction of miltefosine with several drugs, including antifungals and lipid inhibitor, using a checkerboard microdilution method. The drug concentrations ranged from 0.001 to 8.0 μg/ml for miltefosine, 0.03 to 2.0 μg/ml for posaconazole, 0.0007 to 0.5 μg/ml for voriconazole, 4.0 to 256.0 μg/ml for caspofungin, 0.06 to 4.0 μg/ml for amphotericin B, and 2.0 to 128 μg/ml for myriocin. The plates were incubated at 37°C during 48 h. The MIC endpoint was 100% growth inhibition. The interaction was quantitatively evaluated by determining the fractional inhibitory concentration index (FICI): FICI = (MIC miltefosine in combination/MIC miltefosine) + (MIC clinical drug in combination/MIC clinical drug). The FICI was calculated for all possible combinations of different concentrations (107). Interaction curves were also constructed. The interaction between these drugs was classified as synergic at FICI of ≤0.5, indifferent at 0.5 < FICI ≤ 4.0, and antagonistic at FICI of >4.0 (57).

### Construction of A. fumigatus mutants.

To generate the SmiA-3×HA mutant, a 2.9-kb fragment encompassing the *smiA* open reading frame (ORF) and the 5′untranslated region (UTR), along with the 1-kb 3′UTR DNA sequence, were PCR amplified from CEA17 genomic DNA (gDNA) with primer pairs P1/P2 and P4/P5, respectively. The 0.8-kb linker-3×HA-trpC fragment was amplified from the pOB430 plasmid with primers P10/P11, and the *prtA* gene was amplified from the plasmid pPTRI with primers P8/P9.

The SmiA-GFP strain was constructed by the amplification of a 2.9-kb fragment encompassing the *smiA* ORF and the 5′UTR region, along with the 1-kb 3′UTR DNA sequence, by PCR from CEA17 gDNA with primer pairs P1/P3 and P4/P5, respectively. The linker-GFP-trpC fragment was amplified from the pOB435 plasmid with primers P10/P11, and the *prtA* gene was amplified from the plasmid pPTRI with primers P8/P9.

The Δ*smiA* strain was complemented, generating the Δ*smiA*::*smiA^+^* lineage. Specifically, the fragments containing the 5′UTR plus the *smiA* gene, along with the 1-kb 3′UTR DNA sequence, were PCR amplified from CEA17 gDNA with primer pairs P1/P7 and P4/P5, respectively. In addition, these fragments were fused to the *ptrA* gene, which was previously PCR amplified from plasmid pPRTI (primers P8/P9).

All DNA cassettes (smiA^+^::prtA, smiA::GFP::prtA, and smiA::3×HA::prtA) were constructed by *in vivo* homologous recombination by using S. cerevisiae (108). Briefly, the set of fragments of each of the constructions, along with the plasmid pRS426 digested with BamHI/EcoRI, were transformed into the S. cerevisiae SC9721 strain. Whole cassettes of smiA^+^::prtA, smiA::GFP::prtA and smiA::3×HA::prtA were transformed into the *ΔsmiA* strain. Candidates were selected by resistance to pyrithiamine and further verified via Western blotting, reversal of miltefosine sensitivity phenotype, and/or protein functionality.

Primers used in this work are listed in the Table S6 at https://doi.org/10.6084/m9.figshare.14762991.v4. Additionally, the mutant strains constructed in the current work were performed into the background of the Δ*smiA* strain. Positive candidates were selected in the presence of pyrithiamine, purified through three rounds of growth on plates, submitted to gDNA extraction, and confirmed by PCR.

### Protein extraction and immunoblot analysis.

A total of 1 × 10^6^ conidia/ml of each strain was inoculated in 50 ml of Vogel’s medium and grown at 37°C for 16 h under agitation. Mycelia were then washed with RPMI 1640 medium and incubated in RPMI 1640 containing 12.5 μg/ml miltefosine at 37°C for 0, 4, and 8 h at 37°C with shaking. For protein extraction, mycelia were ground into liquid nitrogen and resuspended in 0.5 ml of lysis buffer (10% [vol/vol] glycerol, 50 mM Tris-HCl pH 7.5, 1% [vol/vol] Triton X-100, 150 mM NaCl, 0.1% [wt/vol] SDS, 5 mM EDTA, 50 mM sodium fluoride, 5 mM sodium pyrophosphate, 50 mM glycerophosphate, 5 mM sodium orthovanadate, 1 mM phenylmethylsulfonyl fluoride [PMSF], and 1× complete mini protease inhibitor [Roche Applied Science]). Extracts were centrifuged at 16,000 × *g* for 20 min at 4°C. The supernatants were collected, and the protein concentrations were determined using the Bradford assay (Bio-Rad). Next, 30 μg of total protein extract from each sample was resolved in 10% (wt/vol) SDS-PAGE and transferred to a nitrocellulose membrane for a Western blot assay. Monoclonal anti-HA antibody (Sigma-Aldrich) was used to confirm SmiA-3×HA expression. In addition, anti-α-actin antibody was used to normalize protein loading. The primary antibodies were detected using a horseradish peroxidase (HRP)-conjugated secondary antibody raised in mouse (Sigma-Aldrich). Chemiluminescent detection was achieved using an ECL Prime Western blotting detection kit (GE Healthcare). To detect these signals on blotted membranes, the ECL Prime Western blotting detection system (GE Healthcare, Little Chalfont, UK) and LAS1000 (Fujifilm, Tokyo, Japan) were used.

### Real-time PCR analysis.

Total cellular RNA was extracted using TRIzol reagent (Invitrogen, Life Technologies, Camarillo, CA, USA). Further, RNA was submitted to DNA digestion with RQ1 RNase-free DNase (Promega, Fitchburg, WI, USA) according to the manufacturer’s instructions. The cDNA synthesis was performed by the ImProm-II reverse transcription system (Promega) and oligo(dT). The real-time PCR was performed using the ABI 7500 Fast real-time PCR system (Applied Biosystems, Foster City, CA, USA) and the SYBR green PCR master mix kit (Applied Biosystems) according to the manufacturer’s instructions. Analyses were carried out using three independent biological replicates. The mRNA quantity relative fold change data was calculated using standard curves (109) and normalized by the expression levels of the housekeeping β-tubulin gene. Primer sequences used in this study are listed in Table S6 at https://doi.org/10.6084/m9.figshare.14762991.v4.

### RNA purification and preparation for RNA-seq.

A total of 10^6^ spores/ml of A. fumigatus WT and Δ*smiA* strains were inoculated in 50 ml of Vogel’s medium and grown at 37°C for 16 h under agitation. The suspensions were centrifuged and washed with phosphate-buffered saline (PBS). Mycelia were suspended in VMM containing glucose supplemented with 3 μg/ml miltefosine, or in the absence of any drug, and incubated at 37°C for an additional 30 min. Total RNA was extracted by the TRIzol method. Subsequently, 10 μg of total RNA was subjected to RNA purification using DNase I (New England Biolabs Inc.), and the quality was checked on 2% agarose gel and verified using an Agilent Bioanalyzer 2100 (Agilent Technologies). RNAs selected for further analysis had a minimum RNA integrity number (RIN) value of 8.0. One microgram of purified RNAs was used for library preparation using an Illumina NEBNext Ultra directional RNA library prep kit according to the manufacturer’s protocol and sequenced using the Illumina HiSeq2500 platform at the Genomics and Single-Cell Analysis Core facility at the University of Macau. The expression levels were calculated in reads per kilobase per million and for the differential expression analysis a log_2_ fold change of −1 ≤ log_2_FC ≥ 1 was applied to capture a minimum of 2 times perturbation on the expression levels, with a *P* value of <0.005 and a false discovery rate (FDR) lower than 0.05.

### Chromatin preparation.

A similar experimental design used for the RNA-seq was used for the ChIP-seq experiments. After growth, the cultures were added to 1% formaldehyde for 20 min with gentle shaking at room temperature, and then a final concentration of 0.5 M glycine was added for further incubation for 10 min. The mycelia were collected by filtering and washed with cold water. The cross-linked mycelia were frozen in liquid nitrogen and frozen dried for 2 h before lysis. The cell lysis was processed 6 times beating for 3 min with an ∼100-μl volume of silica beads using a Bullet Blender (Next Advance) with 3 min of cooling between each cycle. Chromatins were extracted as described previously (110) and sonicated using the Qsonica Q800R at 100% amplitude with 10-s on and 15-s off cycles for a total sonication time of 30 min. Chromatin concentration and size (100 to 500 bp) were checked on 2% agarose gel, and the prepared chromatins were stored at −80°C until use.

### Chromatin immunoprecipitation and sequencing library preparation.

Immunoprecipitation was carried out using anti-HA antibody as described previously (111). Immunoprecipitated materials were purified using a Qiagen PCR cleanup kit, and multiplexed sequencing libraries were prepared as described previously (111) using a NEBNext Ultra II DNA library prep kit for Illumina according to the manufacturer’s protocol. Libraries were checked and quantified using DNA high-sensitivity bioanalyzer assay, mixed in equal molar ratios, and sequenced using the Illumina HiSeq2500 platform at the Genomics and Single-Cell Analysis Core facility at the University of Macau.

### Data mapping and bioinformatics analysis.

Raw sequencing reads of ChIP-seq experiments were quality checked using FastQC (http://www.bioinformatics.babraham.ac.uk/projects/fastqc/) and aligned to the Af293 reference genome (genome version s03-m05-r06) using Bowtie2 (version 2.2.9) (112). For peak calling, MACS2 was applied. To determine the presence of conserved miA DNA binding motifs, we carried out a MEME-ChIP analysis to search the 500-bp region surrounding the peaks identified in our ChIP-seq data (http://meme-suite.org).

### Lipid analysis.

A total of 10^6^ spores/ml of A. fumigatus wild-type and Δ*smiA* strains were inoculated in 50 ml of Vogel’s medium and grown at 37°C for 16 h under agitation. The suspensions were centrifuged and washed with PBS. Mycelia were suspended in VMM containing glucose supplemented with 3 μg/ml miltefosine, or in the absence of any drug, and incubated at 37°C for an additional 4 h. Prior to cell lysis, C17-sphingolipids were added to the samples (113, 114). Mandala extraction was carried out as described previously (115), with a few modifications. To facilitate the disruption of mycelia, the samples were vortexed and sonicated for 2 min in the presence of 0.2 g of glass beads. The samples then were submitted to Bligh and Dyer extraction (116). A quarter of each sample obtained from the Bligh and Dyer Extraction was reserved for inorganic phosphate (P_i_) determination, so the relative sphingolipid signal was normalized by the P_i_ abundance. The organic phase was transferred to a new tube and submitted to alkaline hydrolysis of phospholipids (117). Finally, the organic phase was dried and used for mass spectrometry analysis (114).

### Statistical analysis.

Grouped column plots with standard deviation error bars were used for representations of data. For comparisons with data from wild-type or control conditions, we performed one-tailed, paired *t* tests or one-way analysis of variance (ANOVA). All statistical analyses and graphics building were performed by using GraphPad Prism 5.00 (GraphPad Software).

### Fluorescence microscopy.

A total of 10^5^ spores of each strain was inoculated on coverslips in 4 ml of MM for 16 h at 30°C. Coverslips with adherent germlings were left untreated or treated with miltefosine for different periods of time, as indicated. Staining procedures included (i) 5 min of incubation in a solution with propidium iodide (PI; 0.05 mg/ml; Sigma-Aldrich); (ii) 5 min of incubation in a solution with MitoTracker Deep Red FM dye (250 nM) (Invitrogen); and (iii) 10 min of incubation in a solution containing Hoechst 33342 dye (20 μg/ml; Molecular Probes, Eugene, OR, USA). Further, the coverslips were rinsed with PBS (140 mM NaCl, 2 mM KCl, 10 mM NaHPO4, 1.8 mM KH_2_PO_4_, pH 7.4). Slides were visualized on the Observer Z1 fluorescence microscope using a 100× oil immersion lens objective. Differential interference contrast (DIC) images and fluorescent images were captured with an AxioCam camera (Carl Zeiss) and processed using AxioVision software (version 4.8). In each experiment, at least 50 germlings were counted. For GFP and the fluorescent miltefosine analogue MT-11C-BDP, the wavelength excitation was 450 to 490 nm, and the emission wavelength was 500 to 550 nm. For MitoTracker Deep Red FM, the wavelength absorbance/emission was about 644/665 nm. For Hoechst (4,6-diamidino-2-phenylindole) staining, the excitation wavelength was 365 nm and emission wavelength was 420 to 470 nm. For PI, the wavelength excitation was 572/25 nm and emission wavelength was 629/62 nm.

### Virulence analysis in Galleria mellonella model.

The Galleria mellonella larvae were obtained by breeding adult larvae (118) weighing 275 to 330 mg. The larvae were kept in starvation in petri dishes at 37°C in the dark for 24 h prior to infection. The larvae used for the experiment were in the sixth stage of development. For infection, fresh spores from each strain (mutants and wild type) were used. The spores of each strain were counted using a hemocytometer. The stock concentration of spore suspensions used for infection was 2 × 10^8^ conidia/ml, and from this stock, 5 μl was used for larval infection (1 × 10^6^ conidia/larva). The control group was composed of larvae inoculated with 5 μl of PBS to observe any possible death caused by physical trauma. The inoculum was performed using a Hamilton syringe (7000.5KH), and the conidia were inoculated into the lower left proleg of the larvae. After 30 min of the larvae being infected, treatment with miltefosine (M5571; Sigma-Aldrich) was carried out. The drug was rehydrated in distilled water as recommended by the manufacturer (84). The concentrations used for the treatments were 40, 60, and 80 mg/kg of larvae, and each larva was weighed individually and the volume was adjusted to the preestablished concentrations. As a control for the treatments, we made three groups of larvae in which concentrations of 40, 60, and 80 mg/kg were injected. The treatments were also injected into the lower right proleg of the larvae. After infection, the larvae were kept at 37°C in petri dishes in the dark and scored daily. Larvae were considered dead due to lack of movement in response to touch. The viability of the inoculum administered was determined by plating a serial dilution of the conidia in YAG medium. The statistical significance of the comparative survival values was calculated using the log rank analysis of Mantel-Cox and Gehan-Brestow-Wilcoxon by using the statistical analysis package Prism (119).

### Data availability.

The data sets generated for this study are available on request to the corresponding author.
